# Thrombocytopenia induces multiple intracranial hemorrhages in patients with severe burns: A review of 16 cases

**DOI:** 10.3892/etm.2013.1081

**Published:** 2013-04-29

**Authors:** JIANDA ZHOU, JINYAN LIU, CHENGQUN LUO, FENG HU, RUI LIU, ZIZI CHEN, YAO CHEN, WU XIONG, JIANFEI XIE, QUANYONG HE, CHAOQI YIN, SHAOHUA WANG, YANWEN ZHANG, SAINAN ZENG

**Affiliations:** The Third Xiangya Hospital of Central South University, Changsha, Hunan 410013, P.R. China

**Keywords:** burn, multiple intracranial hemorrhages, platelet, coagulation abnormalities, complication

## Abstract

The aim of this study was to explore the etiology and diagnosis of multiple intracranial hemorrhages (ICHs) following severe burns, with a retrospective review of 16 cases of severe burns further complicated by multiple ICHs. Using cranial CT scans of the brains, we identified that all patients presented with low platelet counts and coagulation abnormalities prior to intracranial hemorrhaging. Following conventional treatment and various supporting treatments, five cases succumbed following a progressive reduction in blood platelet levels and the ICHs were cured in 11 cases following the restoration of normal platelet levels. We conclude that low platelet counts and coagulation abnormalities may cause multiple ICHs following severe burns and early diagnosis and treatment is the key to successful treatment.

## Introduction

A burn is defined as damage to body tissue by heat, chemicals, electricity, sunlight or radiation. Scalds from hot liquids and steam, building fires and flammable liquids and gases are the most common causes of burns. Another type of burn is an inhalation injury, caused by inhaling smoke. There are three types of burns: first-degree burns which only damage the outer layer of skin, second-degree burns which damage the outer layer and the layer beneath, and third-degree burns, which damage or destroy the deepest layers of skin and the tissues underneath. Burns may cause swelling, blistering, scarring, shock and mortality. They may lead to infections due to the fact that the skin’s protective barrier is damaged (1). Antibiotics are capable of preventing or treating infections. An intracranial hemorrhage (ICH) is a hemorrhage, or bleeding, within the skull. Intracranial bleeding occurs when a blood vessel within the skull ruptures or leaks. It may result from physical trauma (such as a head injury) or have a non-traumatic cause (as occurs in a hemorrhagic stroke) such as a ruptured aneurysm. Anticoagulant therapy, used in the treatment of disorders of blood clotting, may heighten the risk of intracranial hemorrhage (2). Intracranial hemorrhaging is a serious medical emergency as the build-up of blood within the skull may cause the intracranial pressure to increase, which may crush delicate brain tissue or limit its blood supply (3). Severe increases in intracranial pressure may lead to brain herniation, in which sections of the brain are squeezed past structures in the skull. Multiple ICHs are rare in severe burn patients and the pathogenesis remains unclear. Computed tomography (CT) scans are a definitive tool for accurately diagnosing ICHs (4). Medical treatment and surgical therapy are available for ICH patients. According to the condition of the patient, timely diagnosis and an optimal choice of treatment methods are vital in order to achieve a successful outcome. In recent years, the incidence of burns complicated by intracranial hemorrhaging has rarely been mentioned in the literature. In order to identify disease features and the probable etiology and improve the prevention, diagnosis and treatment of multiple ICHs following severe burns, we reviewed 16 cases from a total of 397 patients with severe burns complicated by multiple ICHs who were admitted to the Third Xiangya Hospital of Central South University (Changsha, China) from 1999 to 2010.

## Patients and methods

### General conditions

This study included 16 patients (5 females and 11 males), ranging from 30–56 years old (mean 45±6.70 years). All 16 patients were treated at a local hospital prior to being admitted to the Third Xiangya Hospital of Central South University. Of these patients, six were admitted to the hospital 7 days after having received their burn injury, four on day eight, four on day 12, one on day 18 and one on day 23. Upon admission, all 16 patients were conscious and not in shock. Cranial CT scans of the brain revealed no abnormalities. The total body surface area (TBSA) of the burn wound was 60–70% in eight cases, 70–80% in four cases, 80–90% in three cases and 95% in one case, an average of 70.5±10.64%. The area of third-degree burns was <15% in ten cases, 15–20% in three cases, 20–30% in two cases, and 30% in one case, with an average of 18.38±7.01%. The study was approved by the Ethics Committee of The Third Xiangya Hospital of Central South University. Written informed consent was obtained from all patients.

### Hemorrhage following severe burns

Emergency CT examination, due to the sudden onset of symptoms in the nervous system in all patients, confirmed intracerebral hemorrhaging. On the eighth day following the burn, eight patients presented with hemorrhaging, two patients presented with hemorrhaging on day nine, three on day 15 and one each on days 20, 25 and 37. There were five cases of brainstem hemorrhaging, nine cases of intracerebral hemorrhaging and two cases of subarachnoid hemorrhaging. The volume of blood lost was <30 ml in ten cases and >30 ml in six cases.

### Dynamic changes in blood platelet levels prior to and following cerebral hemorrhaging

One day prior to cerebral hemorrhaging, the platelet levels reduced in all patients to 29–78 × 10^9^/l (43.7±9.8 ×10^9^/l on average). Following treatment, five patients exhibited a progressive decrease in platelet levels and succumbed. The other 11 cases recovered to an average platelet level of 7.5±1.6 × 10^9^/l.

### Clinical treatment and results

Following admission, all patients received conventional treatment, such as anti-infection, nutritional support and the changing of burn dressings. Following the appearance of symptoms of intracerebral hemorrhaging, various supporting treatments were performed according to the bleeding situation, such as reducing the cranial pressure by fluid restriction, hemostasis, platelet injection, cryotherapy or enrichment with erythrocytes. The final result was that five of the 16 patients succumbed and 11 recovered. No significant difference in intracerebral bleeding was exhibited between the deceased and surviving groups. There was no marked difference (t<1, P>0.05) in the volume of blood lost between the deceased (21.8±15.3 ml) and the surviving groups (19.1±13.01 ml). When the intracerebral bleeding occurred, the platelet count for the deceased group (46.6±12.2 ×10^9^/l) and the survival group (43.3±13.1 ×10^9^/l) also exhibited no significant difference (t<1, P>0.05). The platelet count gradually and steadily reduced in the deceased group following intracerebral bleeding. By contrast, the platelet count increased from the third day following intracerebral bleeding in the surviving group, and within a week the levels had recovered to normal (7.5±1.6 × 10^9^/l).

## Case reports

### Case 1

**Figure 1.**
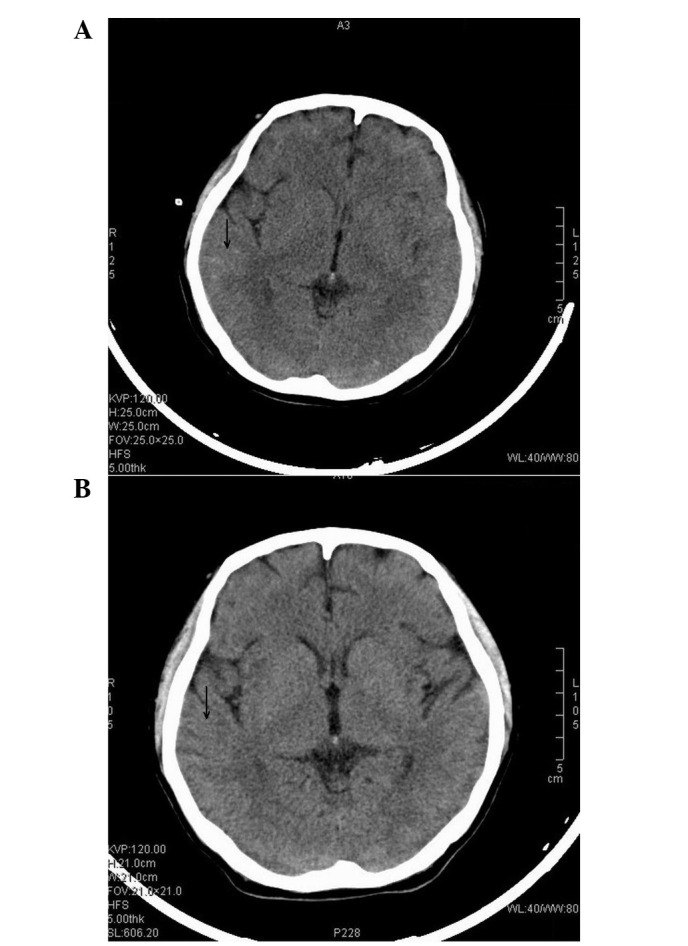
Cranial computed tomography film of Case 1. (A) High-density patchy areas were observed following multiple intracranial hemorrhages. (B) Improvement in hemorrhage following treatment. The arrows in the images indicate the place of bleeding.

A 30-year-old male suffered complete body burns in a boiler explosion. He was admitted to the hospital eight days later and the diagnosis on admission was burns to 95% of the TBSA (3% superficial second-degree burns, 77% deep second-degree burns and 15% third-degree burns) complicated with whole-body burn wound sepsis. The patient had renal failure, anuria, an infection in both lungs, heart failure and severe hypoxemia. The patient received treatment with a ventilator, blood filtration, antibiotics, nutritional support and dressing changes. On day 22 following hospital admission, wound bleeding increased and airway and gastrointestinal bleeding was observed. The platelet levels of the patient were reduced to 41 ×10^9^/l on day 24, giving an activated partial thromboplastin time of 54.3 sec and a plasma prothrombin time of 14.7 sec. The patient was in a light coma with pupillary asymmetry (left pupil 2 mm, right 4 mm). CT revealed multiple patchy areas of high density, particularly in the right temporal lobe. The bleeding lesion was irregular and 2.5 ml in size. There were no abnormalities in the ventricle and cistern. Following symptomatic treatment and fiber bronchoscopy for airway congestion, platelet injection, cryoprecipitation and packed erythrocyte infusion, the patient began to recover on day 29, with a platelet level of 112 ×10^9^/l. On day 43, CT reexamination revealed hematoma absorption (Fig 1). On day 50, the patient exhibited a marked improvement and was transferred to a general ward. The patient received multiple skin grafts and was discharged 3 months later.

### Case 2

**Figure 2.**
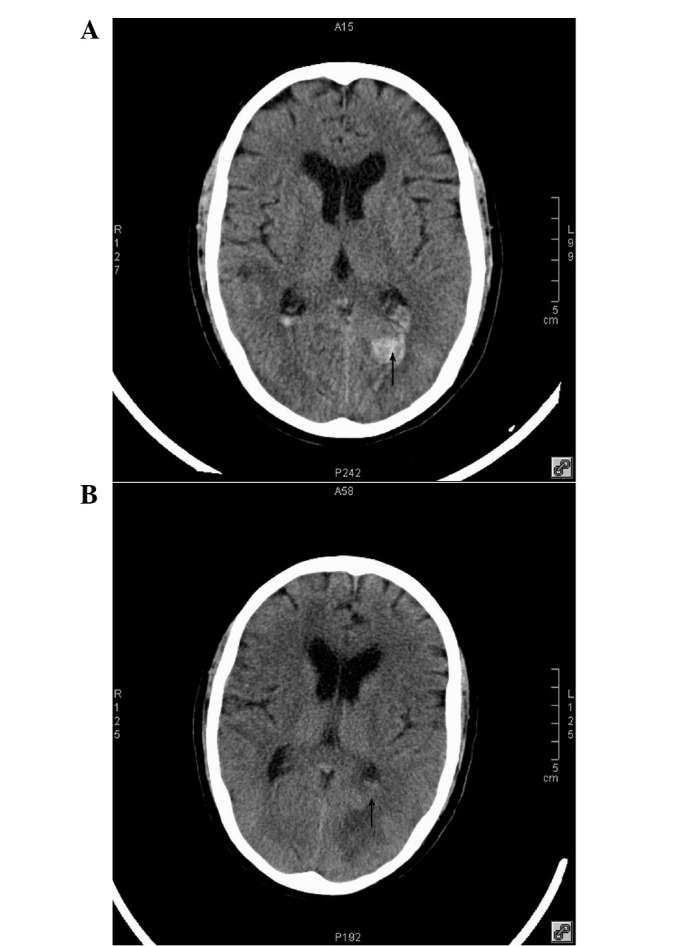
Cranial computed tomography film of Case 2. (A) Multiple regional high-density areas were observed following intracranial hemorrhage. (B) Improvement in hemorrhage following treatment. The arrows in the images indicate the place of bleeding.

A 54-year-old male, was admitted to hospital for a cough and throat pain 23 days after receiving alkali burns to the entire body. The diagnosis on admission was burns to 80% of the TBSA (20% superficial second-degree burns, 30% deep second-degree burns and 30% third-degree burns) complicated by burn wound sepsis and lung infection. Hypoproteinemia, anemia and a severely low platelet count were observed, the patient received wound dressings, escharectomy, antibiotic treatment, packed erythrocyte infusion, platelet injection and nutritional support. On day 12 following admission, the patient complained of a severe headache and dizziness, and his platelet level was found to be 38 × 10^9^/l. The CT revealed cerebral hemorrhaging in the left occipital lobe, subarachnoid hemorrhaging and slight ventricular hemorrhaging. On day 14, a place of bleeding in the right lower limb appeared and CT revealed a new lesion in the right parietal lobe, however, a lumbar puncture revealed that the cerebral spinal fluid was normal. On day 16, the place of bleeding in the right lower limb was aggravated and CT revealed a new lesion in the left parietal lobe with an improvement to the subarachnoid and ventricular hemorrhages. On day 18, the patient fell unconsciousness with intermittent, paroxysmal, generalized tetanic spasms. CT revealed no changes in the left parietal lobe hemorrhage, but reductions in the right occipital lobe, subarachnoid and ventricular hemorrhages were observed. The platelet level was 108 ×10^9^/l. On day 20 the patient regained consciousness, presenting with first-degree muscle strength in the right lower limb. On day 47, CT revealed a significant improvement to the hemorrhage of the left occipital lobe (Fig. 2). Further symptomatic treatments, including wound dressing, antibiotics, decompression and nutritional support, led to the patient being discharged three months later.

## Discussion

Burn injuries are often followed by a profound hypermetabolic response that may last long after the injury was initially sustained (5). The hypermetabolic response is responsible for devastating muscle and protein catabolism, insulin resistance and cardiac dysfunction that may last for months and marked growth retardation which may impede proper development (6). Patients have supraphysiologic metabolic rates, multi-organ dysfunction and increased levels of inflammatory cytokines and acute phase proteins (7). This response may lead to alterations in the consciousness of the burn patients.

Burn patients with sepsis are often mentioned in the literature, but those with burns concurrent with intracranial lesions are often neglected. Cho *et al* reported a study of patients who had strokes following burns (8). Physical examination is challenging to complete in burns patients. The observation of pupillary changes in patients with facial burns is difficult, as the eyelids undergo serious edema; furthermore, it is not easy to perform a pathological and neurological examination of the facial wounds of a burns patient as they are covered thickly with layers of scar tissue.

Multiple ICHs are rare in severe burn patients and the pathogenesis remains unclear. There have been reports that the consumption of blood platelets, fibrinogen, plasmin and blood coagulation factor by the burned tissue and the disseminated intravascular coagulation (DIC) process may contribute to intracerebral hemorrhaging (9). Cho *et al* (8) hypothesized that wound infection and sepsis are the main reasons for shock following a burn. According to the cases we examined, intracranial hemorrhaging occurred during the infection period of shock. All patients had serious wound sepsis, with a low platelet count and blood coagulation disorders prior to the intracerebral hemorrhaging. Five patients (cases 12–16) succumbed; despite the administration of a platelet infusion, their platelet counts had been progressively decreasing. In the other 11 surviving cases, the blood platelet count rose slowly following treatment with a platelet infusion and blood filtration. Therefore, we believe that the direct cause of multiple intracranial hemorrhaging in patients with severe burns is a sharp decline in the platelet count and blood coagulation disorders, and its indirect causes are systemic sepsis and wound sepsis. As the patients were hospitalized in hospitals with insufficient medical facilities, we consider that the informal treatment and medication may be the cause of wound infection and sepsis, indirectly leading to intracerebral hemorrhaging.

Patients with a large burn area present with thrombocytopenia and blood coagulation disorders easily. The rapid consumption of platelets is a concern, but the main concerns in the middle-late stage are serious infections and the direct absorption of toxins leading to the rapid decline of platelet levels.

Inflammation is a defense mechanism against damaging factors such as severe trauma and infection which may activate blood coagulation leading to blood clotting and microcirculation disorders and even organ dysfunction (8). However, the mechanism is complex and the correlation between the two remains unclear (10). During an inflammatory reaction, inflammation mediators are released which activate blood coagulation and consume mass clotting factors through the ‘waterfall sample cascade’ which may lead to blood coagulation disorders (11–13). In serious wounds, bacteria and external toxins are absorbed into the bloodstream; in the current study, *Staphylococcus aureus* was cultured from the surface of the wound in six cases and from blood cultures in two cases . This stimulates the release of a variety of inflammation mediators, causing a marked increase in platelet adhesion and the clumping together of platelets to form large numbers of tiny white thrombuses in the circulation when blood coagulation increases. The tiny blood clots cause a series of pathological physiology reactions such as thrombocytopenia, a reduction in fibrinogen, an increase in the activity of dissolved fibrinogen, serious bleeding and coagulant function failure. Six cases exhibited level I coagulant function failure the day prior to intracerebral haemorrhaging, and two cases who succumbed had level II coagulant function failure (14). When coagulant function failure occurs in patients with serious burns, it is preceded by gastrointestinal and skin mucous membrane bleeding. Therefore, when platelets are being infused and the filter is not capable of maintaining the platelet levels, there is a risk of intracranial hemorrhaging.

In certain patients, the decision to conduct an emergency CT examination was made due to the sudden onset of clinical symptoms such as headache and hemiplegia. Diagnosis was determined according to clinical manifestations such as an altered mental state and a pupillary change, CT scans and laboratory data. The majority of patients with burns appear to have a sudden alteration to their mental state when their symptoms improve; when symptoms such as severe headache, nausea and vomiting appear in patients, this is a warning sign. Head CT examinations established the amount of bleeding and whether the blood loss was stable; this allowed us to decide whether to continue with a more conservative treatment or to proceed with surgery according to the specific circumstances of each patient. In CT imaging, shadowing due to increased flake density (Fig. 1) or focal density (Fig. 2) is common.

In a previous study, we examined stroke diagnoses in elderly burn patients (15). Burns causing intracerebral hemorrhaging appear initially with the bleeding of skin mucous membranes and gastrointestinal and internal organs prior to intracerebral hemorrhaging; therefore we should pay particular attention to severe wound bleeding, hematemesis or blood in the stools. During the burn infection period, the platelet count should be checked often as a sudden drop in the platelet level is a useful diagnostic indicator of systemic infections. For simple stroke patients, a marked reduction in the platelet levels and skin mucous membrane and internal bleeding are not generally observed prior to intracranial hemorrhaging.

For burn patients with intracranial hemorrhaging, prevention is preferable to treatment, as the long-term use of antibiotics may cause double fungal infections which may lead to increased morbidity and mortality (16). In general, strategies to prevent infection, such as early excision and grafting, aggressive anti-microbial therapy, including the use of colistin, and early enteral feedings improve the survival rate (17–18). In the current study, two the of 16 cases underwent early escharectomies, leading to a significant improvement in body and wound infections. Treatment for intracranial hemorrhaging should be initiated as soon as the diagnosis is confirmed, with platelet infusions, hemostasis, decompression, blood filtration, antibiotics and nutritional support. Key to successful treatment are controlling the platelet levels via hemostasis, non-heparinized blood filtration and effective anti-inflammatory treatment to prevent platelets counts from dropping further.

The role of surgical treatment for these patients requires further study. Conservative treatment is usually recommended due to the fact that treating sporadic or multiple hemorrhages with surgery is complex. We performed conservative treatment in all sixteen cases, with success in eleven cases (68.75% success rate). Large hemorrhages or unlimited bleeding require surgery to clean the intracranial edema and reduce intracranial pressure: for basal ganglia hemorrhage >30 ml, surgery should be timely and a minimally invasive intraoperative puncture hematoma or a small bone window to clear hematoma should be selected; for cerebellar hemorrhage >10 ml or combined with hydrocephalus, surgery may be considered; if the lobes are bleeding then conservative treatment should be provided.

The incidence of particularly severe burns accompanied by multiple intracranial hemorrhaging is not high. In recent years, it has been rarely mentioned in the literature. Although the patient survival rate for severe burns patients also undergoing multiple intracranial hemorrhaging is low and treatment costs are high, if the patients are provided with sufficient attention and positive symptomatic treatment then there is a good chance of a successful treatment.
